# Phytochemical Profiling and Pharmacological Evaluation of Methanolic Leaf Extract of *C. digyna* for Cytotoxic, Anti‐inflammatory, Antioxidant, Antiarthritic, and Analgesic Activities

**DOI:** 10.1002/fsn3.4504

**Published:** 2024-11-04

**Authors:** Kanij Fatema, Md. Abdur Rashid Mia, Tanzina Sharmin Nipun, S. M. Moazzem Hossen

**Affiliations:** ^1^ Department of Pharmacy University of Chittagong Chittagong Bangladesh; ^2^ Northern Marianas College CNMI USA

**Keywords:** acetic acid‐induced writhing, brine shrimp lethality bioassay, *Caesalpinia digyna*, carrageenan‐induced paw edema, DPPH assay, HRBC membrane lysis, protein denaturation, tail immersion

## Abstract

*Caesalpinia digyna* (Family: Fabaceae) is traditionally used in Ayurvedic medicine for various medicinal purposes, including as a treatment for wounds, leprosy, skin diseases, fever, diabetes, etc. Although the root and stem of this plant have a significant medicinal value, there was little research on the leaves of this plant. This study aimed to investigate the qualitative phytochemical profile and evaluate the in vitro cytotoxic, anti‐inflammatory, antioxidant, and antiarthritic activities, as well as the in vivo anti‐inflammatory and analgesic activities, of *C. digyna* leaf extract. The methanolic extract of *C. digyna* leaves was prepared using an ultrasonic‐assisted extraction process. In vitro and in vivo anti‐inflammatory activities were evaluated using the hypotonicity‐induced hemolysis and carrageenan‐induced paw edema methods, respectively. Additionally, the extract was assessed for in vitro DPPH (1, 1‐diphenyl‐2‐picrylhydrazyl) free radical scavenging, antiarthritic (protein denaturation), and in vivo analgesic (acetic acid‐induced writhing and tail immersion) activities. Brine shrimp lethality bioassay (BSLB) showed moderate cytotoxic activity (LC_50_ = 2.25 μg/mL) compared with the standard vincristine sulfate (LC_50_ = 1.61 μg/mL). In vitro, anti‐inflammatory activity exhibited 85.13% (IC_50_ value = 2.51 μg/mL) inhibition of Human Red Blood Cell (HRBC) membrane lysis at a concentration of 2000 μg/mL whereas in vivo anti‐inflammatory study exerted its maximum effect (*p* < 0.05) at 400 mg/kg bw dose. This extract also showed significant antioxidant (IC_50_ = 0.218 μg/mL), antiarthritic (83.61% inhibition) activity, and moderate analgesic effect (*p* < 0.05) in both methods. These research findings indicated that *C. digyna* leaves extract has potent antioxidant, analgesic, and anti‐inflammatory effects which can be used as a supplementary medication for inflammatory pain‐relieving factors. In future, finding the mechanism involved in these effects could have significant impact on clinical science.

## Graphical abstract

DPPH: 1, 1‐diphenyl 2‐picrylhyorazyl, HRBC: human red blood cell.

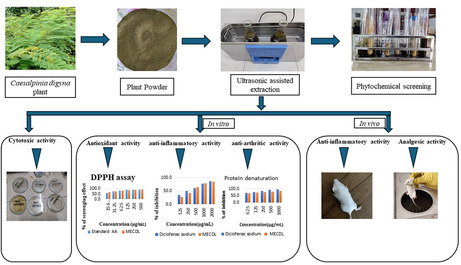

## 1 Introduction

Medicinal plants have been used worldwide since ancient times. About 85% people of in the world depend on plant‐derived medication (Fitzgerald, Heinrich, and Booker 2019). In Bangladesh, most people use medicinal plants for their primary treatment purposes, especially by rural people as there are limited healthcare facilities in our country. Therefore, isolating medicinal compounds from plants and evaluating the pharmacological properties of plants are becoming more popular (Ahmed et al. 2021).

The medicinal plant contains a variety of compounds that give different pharmacological actions namely antioxidant, anti‐inflammatory, antiarthritic, analgesic, and so on. Before conducting any pharmacological tests, the toxicity of the extract should be measured to understand whether it is safe for cells or not. This will ease the determination of dose for experiments (van Wyk and Prinsloo 2020).

Free radicals are chemical compounds that have an unpaired electron in their orbit which causes it to be very reactive. It plays an important role in cellular damage and leads to inflammation (Geronikaki and Gavalas 2006). Inflammation is an immunological response of living tissue to injurious stimuli, like pathogens, irritants, or damaged cells which is characterized by swelling, pain, and redness (Chen et al. 2018). However, inflammation is a body's natural immunological response but its uncontrolled stage can damage the body's system. Inflammation activates different types of cells which secrete various inflammatory markers such as prostaglandin (PGE2), cytokines (TNF‐α, IL‐1β, IL‐6), and nitric oxide (NO) (Woranam et al. 2020). If the inflammation is persistent, it will lead to rheumatoid arthritis (RA). RA is an immune‐mediated disease that causes bone deformation, joint swelling, and pain (Tatiya et al. 2017). To reduce inflammation and pain relief, NSAIDs (indomethacin, ibuprofen) are used whereas both acute and chronic inflammation can be treated by non‐opioid analgesics (aspirin) (Nunes et al. 2020). Anti‐inflammatory agents are also used in RA patients as continuing therapy (Pawar et al. 2019). The extended use of these medications causes harmful effects such as respiratory depression, digestive problems, kidney damage, and even dependence. Therefore, researchers are now trying to find new analgesic and anti‐inflammatory agents devoid of those effects (Tatiya et al. 2017). Nowadays research is going on to discover novel therapeutic substances from medicinal plants that could be used to suppress inflammation (Vadivu and Lakshmi 2008).

*Caesalpinia digyna* is a medicinal plant of the family Fabaceae which was chosen for this research purpose. The plants of this family are renowned for their traditional use such as asthma, cold, cough, skin disease, and abscess (Choi et al. 2006). Prior root and stem extracts are reported to exhibit antioxidant, anti‐tubercular, anti‐hypertensive, anti‐inflammatory, antipyretic, hypoglycemic, and hypolipidemic activity. Some in silico studies are carried out for the leaves of this plant (Muhammad et al. 2019). So, there is a chance to have the same type of activities in the leaves of *C digyna*. It is one of the most common medicinal plants grown in the Indian subcontinent and it is also available in Bangladesh. Chemical examination of *C. digyna* illustrates the presence of different phytochemicals such as Caesalpinine C, Caesalpinine A, methoxybonducelline, bonducellin, isobonducellin, intricatinol, isointricatinol, e‐eucomine, and z‐eucomine (Emon et al. 2021).

The aim of this study is to investigate the phytochemical profile, cytotoxic activity, and in vitro and in vivo study of the anti‐inflammatory activity of methanolic extract of *C. digyna* leaves.

## 2 Material and Methods

### 2.1 Sample Collection and Preparation

The leaves of *C digyna* were collected from the Hazarikhil Wildlife Sanctuary of Chittagong, Bangladesh, and identified by taxonomist Dr. Shaikh Bokhtear Uddin, University of Chittagong, Bangladesh. The leaves were washed with purified water and dried in a shaded area for a duration of 14 days at a temperature of 28°C and a relative humidity of 86%. Then leaves were ground into a fine powder using a 1000 W Panasonic mixer grinder. After that, it was stored in the refrigerator in an airtight container for further analysis.

### 2.2 Preparation of *C. digyna* Leaves Extract

A quantity of about 50 g of the powder was placed in a pristine glass beaker and immersed in 1.5 L of methanol, with a ratio of 1 part powder to 30 parts methanol. Then the beaker was sealed with foil with holes and sonicated for 30 min at 40°C (Nipun et al. 2020). The whole mixture was then filtered through filter paper (Whitman No. 1) followed by the removal of the solvent using a Buchii Rota evaporator at 60°C temperature and at a speed of 80 rpm. The extract's yield was quantified using the following formula:

$$\text{Yield of extract}\;\left(\%,w/w\right)=\left({\mathrm{wt}}_{b}/{\mathrm{wt}}_{a}\right)\times 100\%$$

wt_a_ = initial weight of the dried leaves powder; wt_b_ = final weight of the dried extract.

### 2.3 Phytochemical Screening

Qualitative phytochemical screening was conducted using the following standard procedures: the ferric chloride test and lead acetate test for phenols; Wagner's test and Mayer's test for alkaloids; zinc hydrochloric acid reduction test and lead acetate test for flavonoids; Salkowski test for triterpenes; Libermann–Burchard test and Salkowski reaction test for steroids; acetone‐water test for resins; sodium hydroxide reagent test for glycosides; Keller–Killiani test for glycosides; shake or foam test for saponins; general test for resins; Fehling's test for reducing sugars; Molisch's test for carbohydrates; ferric chloride test and lead subacetate test for tannins; Biuret test and xanthoproteic test for proteins; and general test and spot test for fats and fixed oils (Abubakar and Haque 2020).

### 2.4 Cytotoxicity

The cytotoxic activity of the extract was evaluated using the BSLB method, as published by Choudhari and Lokhande (2023). Initially, the extract was made at different concentrations (1, 0.5, 0.25, 0.125, 0.625, and 0.313 mg/mL) whereas vincristine sulfate was employed as a positive control at various doses (50, 30, 10, 5, and 2.5 μg/mL). Dimethyl sulfoxide (DMSO) was used as a solvent to prepare the standard and experiment sample. DMSO was also considered as the negative control. In a Petri dish, 5 mL sea water was taken and ten living nauplii were added to each dish using a pipette. Different concentrations of sample and standards were applied to each dish which was incubated for 24 h in optimum conditions (Temperature: 25°C ± 2°C, 12 h light). A magnifying glass was used to calculate the number of nauplii (survived) in each dish after 24 h. The mortality rate was calculated by dividing the number of dead nauplii by the initial number of living nauplii and then multiplying the result by 100.

Then the percentage of death is calculated followed by plotting a probit analysis graph to determine the LC_50_ value using the software “Microsoft Office 365‐excel.”

### 2.5 In Vitro

#### 2.5.1 Anti‐Inflammatory Effect

The anti‐inflammatory properties of the extract were evaluated by the hypotonicity‐induced hemolysis method of Yesmin et al. (2020). Various concentrations (2000, 1000, 500, 250, and 125 μg/mL) of leaf extract and positive control (diclofenac sodium) were prepared based on previous studies and placed in separate test tubes. Subsequently, 0.5 mL of HRBC suspension, 1 mL of phosphate buffer, and 2 mL of hypo‐saline were introduced into each test tube. The test tubes were then incubated at a temperature of 37°C for 30 min. Following the incubation period, the tubes were subjected to cooling and centrifugation at a speed of 4000 RPM for 20 min. Afterward, the UV spectrophotometer was used to measure the absorbance of the supernatant fluid at a wavelength of 560 nm using distilled water (DW) as blank.

The following formula was used to calculate the inhibition (%) of protein denaturation:

$$\text{Inhibition}\;\left(\%\right)=\frac{C-S}{C}\times 100$$

where *C* represents the absorbance value of the control and *S* represents the absorbance of the sample.

#### 2.5.2 Antioxidant Activity

The scavenging action of the free radical DPPH was used to assess the antioxidant activity of the leaf extract using the method outlined in Rohmah et al. (2020). A total of 3 mL of a methanol solution containing 0.004% DPPH was combined with 2 mL of a solution of extract at different concentrations (500, 250, 125, 62.5, 31.25, and 15.625 μg/mL). DPPH in methanol and ascorbic acid (AA) were used as control and reference standards, respectively. Subsequently, the absorbance was quantified using a UV spectrophotometer at a wavelength of 517 nm, with methanol serving as a reference solution. This measurement was conducted after a 30 min incubation period at room temperature, in a light‐restricted environment. The following formula was used to get the percentage of inhibition of the free radical DPPH (I%):

$$\left(I\%\right)=\left(1-A_{\text{sample}}/A_{\text{blank}}\right)\times 100$$

where, *A*_sample_ is the absorbance of the test material; *A*_blank_ is the absorbance of the control.

#### 2.5.3 Antiarthritic Activity

The assessment of in vitro antiarthritic activity was conducted utilizing a protein denaturation method with minor modifications described by Joshi, Jat, and Patil (2021). In order to create the test solution (0.50 mL), 0.45 mL of BSA (0.5% w/v) was combined with 0.05 mL of extract containing various concentrations (1000, 500, 250, 125, and 62.5 μg/mL). In contrast, the control solution was prepared using DW in place of BSA. The preparation of the standard solution is the same as the test solution where 0.05 mL diclofenac sodium was replaced with the extract. Again 0.45 mL of BSA was mixed with 0.05 mL of DW to prepare the product control. The pH of all solutions was adjusted to 6.3 using 1 N HCl. The solutions were then subjected to incubation for a duration of 20 min at a temperature of 37°C, accompanied by an additional 3 min at a temperature of 57°C. Finally, the solutions were cooled and then 2.5 mL of phosphate buffer was added to each. After that, a UV–visible spectrophotometer was used to determine the absorbance of the solutions at a wavelength of 416 nm.

The calculation for determining the percentage inhibition of protein denaturation is as follows:

$$\%\;\text{of Inhibition}=100-\left[\left\{\left(A_{\left(t\right)}-A_{\left(\mathrm{pc}\right)}\right)/A_{\left(\mathrm{tc}\right)}\right\}\times 100\right]$$

where, *A*_(t)_ = absorbance of test solution; *A*_(pc)_ = absorbance of product control; *A*_(tc)_ = absorbance of test control.

### 2.6 In Vivo

#### 2.6.1 Experimental Animal

In order to conduct experiments, male albino mice ranging in age from 7 to 8 weeks and weighing (25 ± 5) g were acquired from Rajshahi and subsequently relocated to the Department of Pharmacy (Animal House) at the University of Chittagong. All the mice were kept in the experimental environment (temperature: 26°C ± 2°C; RH: 55%–60%; 12 h dark light cycle) for 4 weeks to adjust to the environment where food and water were available to them in adequate amounts. The research work was approved by the AERB (Animal Ethics Review Board), Faculty of Biological Science, University of Chittagong, Bangladesh with reference number AERB‐FBSCU‐20230731‐(1). After completion of the study, the animals were treated according to AERB guidelines.

#### 2.6.2 Anti‐Inflammatory Activity

The anti‐inflammatory effect was assessed using carrageenan‐induced paw edema, following the methodology reported by Szekalska et al. (2020). Initially, a total of 30 mice were distributed evenly among five groups, with each group consisting of six mice. Following a 12‐h fasting period, all the groups were treated orally as follows: Group I, the negative control group, received a 10 mL/kg bw solution of 1% tween‐80; Group II, the standard group, was given a 100 mg/kg of diclofenac sodium; other three groups administered different concentrations of leaves extract (100, 200, and 400 mg/kg bw). Control, standard, and extract solutions were given orally after 1 h of subcutaneous administration of 100 μL of carrageenan (1% w/v in 0.9% normal saline) into the hind paw of each mouse on the right side to induce inflammation. The paw's circumference was measured at 0, 1, 2, 3, and 4 h using a thread and meter ruler. Changes in paw circumference (mm) before and after carrageenan injection indicated edema in the paw.

#### 2.6.3 Analgesic Activity

The analgesic effectiveness of the extract was evaluated in mice model using the acetic acid‐induced writhing test and the tail immersion test, in accordance with the protocol outlined in Gupta et al. (2015).

##### 2.6.3.1 Acetic Acid‐Induced Writhing Method

At first, Swiss albino mice (male) weighing between 25 and 30 g were separated into five groups, each with six animals, and were given the following treatments orally: group 1 received the standard indomethacin (10 mg/kg bw), group 2 received the control (1% Tween 80 & DMSO in saline), and group 3, 4, and 5 were received extract at doses of 100, 200, and 400 mg/kg bw individually. After 40 min, 0.7% AA (0.1 mL/10g bw) was administered to all the animals intraperitoneally. Then the writhing of each mouse was counted for 15 min after 5 min of acetic acid administration. The following calculation was used to compute the percentage of protection:

$$\%\;\text{Protection}=\frac{N_{C}-N_{T}}{N_{C}}\times 100$$

where, *N*_C_ = no. of writhes in the control group; *N*_T_ = no. of writhes in the test group.

##### 2.6.3.2 Tail Immersion Method

Briefly, a total of thirty mice (male) were split evenly into five groups, with each group consisting of six mice. The tail (5 cm) was immersed in a water bath with a temperature of 55°C ± 0.5°C and the time taken by the mice to withdraw their tail is measured which is known as reaction time, with 20 min cut‐off. The baseline reaction time was measured before treatment (30 min before). Then each group of mice received different concentrations of extract solutions (100, 200, 400 mg/kg bw), Morphine (2 mg/kg bw), and control respectively. Subsequently, response times were assessed at intervals of 30, 60, 90, and 120 min following the therapy. The percentage of elongation was measured by the following equation:

$$\text{Elongation}\;\left(\%\right)=\frac{L_{T}-L_{C}}{L_{T}}\times 100$$

where, *L*_T_ = latency of test; *L*_C_ = latency of control.

### 2.7 Statistical Analysis

The results were presented as mean ± SD. One‐way ANOVA (Tukey test) was employed for statistical analysis using Minitab 21. The significance level was established at *p* < 0.05.

## 3 Result

### 3.1 Extraction Yield and Phytochemical Screening

The extraction yield of the leaves extract was obtained 25%. Phytochemical screening results showed that phenols, alkaloids, flavonoids, triterpenes, steroids, resins, glycosides, carbohydrates, and tannins were present in the extract. On the other hand, Saponins, reducing sugars, Protein, Fats, and fixed oils were absent in the extract. The phytochemicals present in this extract are given in Table 1 along with the method used in the experiment.

**TABLE 1:** Phytochemical screening of the *Caesalpinia digyna* leaves extract.

Secondary metabolites	Name of test	Result
Alkaloids	Wagner's test	+
	Mayer's test	+
Tannins	Ferric chloride test	+
	Lead subacetate test	+
Steroids	Salkowski reaction test	+
	Libermann–Burchard's test	+
Triterpenes	Salkowski reaction test	+
Flavonoids	Zinc hydrochloric acid reduction test	+
	Lead acetate test	+
Saponins	Shake or foam test	−
	Foam test	−
Resins	General test	+
Glycosides	Sodium hydroxide reagent test	+
Cardiac glycosides	Keller–Killiani test	−
Phenols	Ferric chloride test	+
	Lead acetate test	+
Reducing sugars	Fehling's test	−
Carbohydrate	Molisch's test	+
Protein	Biuret test	−
	Xanthoproteic test	−
Fats and fixed oils	General test	−
	Spot test	−

*Note:* (+) = present of phytochemicals and (−) = absent of phytochemicals.

### 3.2 Cytotoxic Activity

**FIGURE 1:**
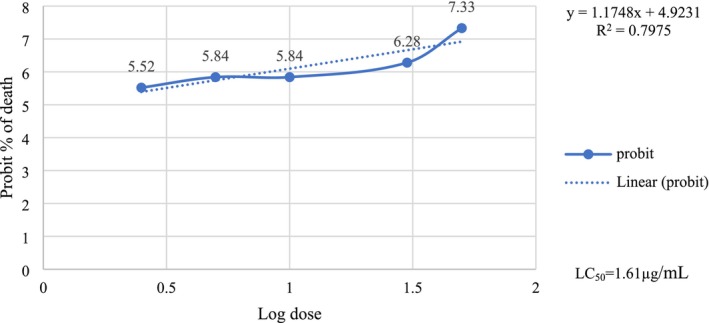
Effect of vincristine sulfate on brine shrimp nauplii.

**FIGURE 2:**
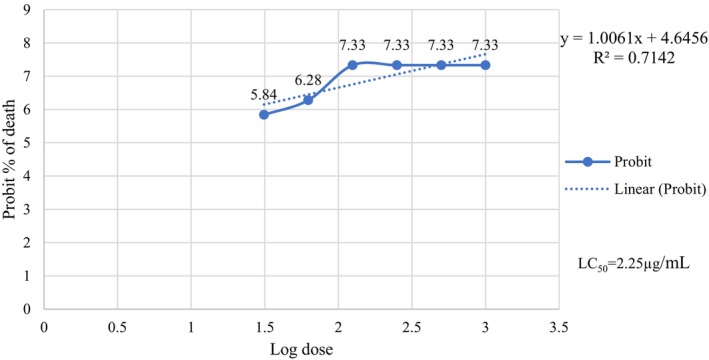
Effect of leaves extract on brine shrimp nauplii.

LC₅₀ refers to the concentration or dose of a toxic substance that results in the death of 50% of the test organisms within a specified exposure period. LC₅₀ values are crucial for determining the toxicity level against specific organisms. The LC_50_ value of the extract was determined by calculating probit from the probit chart using the percentage of mortality preceded by plotting the logarithm of concentration against probit. The positive control was significantly more lethal than the negative control (sea water), with an LC_50_ value of 1.61 μg/mL (Figure 1), while the LC_50_ value for the extract was 2.25 μg/mL (Figure 2).

### 3.3 In Vitro

#### 3.3.1 Anti‐Inflammatory Effect

**FIGURE 3:**
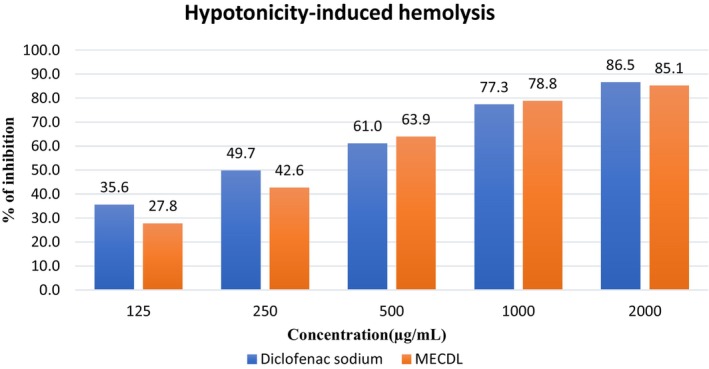
Percentage of anti‐inflammation inhibition vs. concentration of extract and diclofenac sodium.

Figure 3 shows that the extract exhibits the highest inhibition (85.13%) of the HRBC membrane at 2000 μg/mL, whereas the lowest inhibition (27.76%) was shown at 125 μg/mL. *C. digyna* leaves extract showed concentration‐dependent inhibition as the following sequence: 125 < 250 < 500 < 1000 < 2000 μg/mL. The leaf extract has a potent anti‐inflammatory effect with an IC_50_ value of 2.51 μg/mL when compared with diclofenac sodium (IC_50_ value of 2.42 μg/mL).

#### 3.3.2 Antioxidant Activity

**FIGURE 4:**
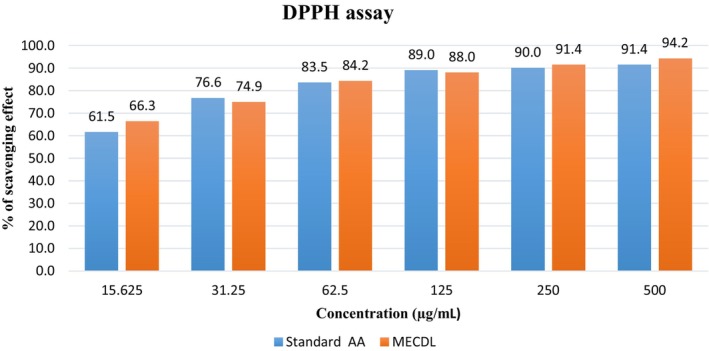
Percentage of scavenging effect vs. concentration of extract and AA.

According to Figure 4, at a concentration of 500 μg/mL, the leaf extract and AA exhibited scavenging effects of 94% and 91%, respectively. The IC_50_ values of the extract and AA were found 0.13 and 0.22 μg/mL respectively.

#### 3.3.3 Antiarthritic Effect

**FIGURE 5:**
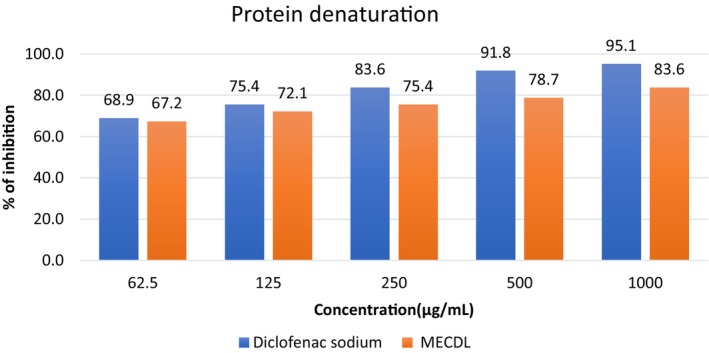
Comparison between percentages of inhibition of protein denaturation on different concentrations of diclofenac sodium and extract solutions.

In this study, leaves extract showed concentration depended on the inhibition of protein denaturation throughout the concentration range from low to high which is depicted in Figure 5. In the comparison of the percentage of protein denaturation inhibition of the extract and standard at 1000 μg/mL concentration, they showed nearly similar percentages which are 83.61% and 95.08%, respectively.

### 3.4 In Vivo Study

#### 3.4.1 Anti‐Inflammatory Activity

The leaves extract significantly attenuated inflammation induced by carrageenan. This effect was dose‐dependent and remained significant up to 24 h after administration as shown in Table 2. The extract at 400 mg/kg bw showed maximum effect after 2 and 3 h. Other doses reduced the inflammation slowly, but 400 mg/kg bw reduced it very quickly.

**TABLE 2:** Effect of MECDL on carrageenan‐induced paw edema in mice.

Animal group	Increase in paw edema thickness, mm. Mean ± SD				
	1 h	2 h	3 h	4 h	24 h
Group‐1 (control)	1.58 **±** 0.15^a^	1.58 **±** 0.15^a^	1.48 **±** 0.12^a^	1.47 **±** 0.14^a^	1.27 **±** 0.08^a^
Group‐2 (indomethacin)	1.52 **±** 0.12^a^	1.27 **±** 0.14^c^	1.10 **±** 0.09^c^	1.03 **±** 0.10^c^	1.03 **±** 0.10^b^
Group‐3 (100 mg/kg bw)	1.67 **±** 0.14^a^	1.52 **±** 0.16^a,b^	1.33 **±** 0.12^a,b^	1.23 **±** 0.10^b^	1.20 **±** 0.14^a,b^
Group‐4 (200 mg/kg bw)	1.50 **±** 0.11^a^	1.37 **±** 0.08^b,c^	1.22 **±** 0.12^b,c^	1.13 **±** 0.10^b,c^	1.13 **±** 0.10^a,b^
Group‐5 (400 mg/kg bw)	1.62 **±** 0.12^a^	1.28 **±** 0.07^c^	1.13 **±** 0.08^c^	1.13 **±** 0.08^b,c^	1.12 **±** 0.07^a,b^

*Note:* Experimental mean values are represented as mean ± SD (*n* = 6). Different letters of the data indicate different significance (*p* < 0.05; a < 0.05, b < 0.01, c < 0.001). For data analysis, Tukey's multiple comparison test was used.

#### 3.4.2 Analgesic Activity

##### 3.4.2.1 Acetic Acid‐Induced Writhing Method

The leaves extract significantly decreased the number of writhes at high doses which is represented in Table 3. In terms of protection from pain, the highest value (52.74%) was observed in group 5 at 400 mg/kg bw among all other concentrations of extract. Furthermore, in the case of standard indomethacin, it was found 65.65% inhibition at the dose of 10 mg/kg bw.

**TABLE 3:** Analgesic activity of the extract by acetic acid‐induced writhing.

Animal group	Number of writhing (mean ± SD)	% of inhibition of writhing
Group‐1 (control)	45.7 **±** 6.72^a^	0
Group‐2 (indomethacin)	15.7 **±** 1.75	65.65
Group‐3 (100 mg/kg bw)	38.3 **±** 3.56^b^	16.19
Group‐4 (200 mg/kg bw)	28.8 **±** 2.58^c^	36.98
Group‐5 (400 mg/kg bw)	21.6 **±** 1.98	52.74

*Note:* Experimental mean values are represented as mean ± SD (*n* = 6). Different letters of the data indicate different significance (*p* < 0.05; a < 0.05, b < 0.01, c < 0.001). For data analysis, Tukey's multiple comparison test was used.

##### 3.4.2.2 Tail Immersion Method

The leaves extract produced 54.62%, 47.35%, 26.37%, and 25.24% elongation of reaction time at 30, 60, 90, and 120 min, respectively, after oral dose administration at 400 mg/kg body weight. At this dosage, the percentage elongation of response time peaked at 30 min (Table 4) (54.62%), which was more in line with the normal dose of morphine (64.2%) at 2 mg/kg bw.

**TABLE 4:** Analgesic activity of leaves extract by tail immersion method.

Animal group	Mean ± SD (% of elongation)				
	Pretreatment	30 min	60 min	90 min	120 min
Group‐1 (control)	3.86 **±** 0.32^a,b^	4.17 **±** 0.49	3.68 **±** 0.29^d^	3.91 **±** 0.66^c^	3.11 **±** 0.47^c^
Group‐2 (morphine)	3.17 **±** 0.20^b^	11.65 **±** 0.69^a^ (64.2%)	10.06 **±** 1.02^a^ (63.42%)	10.01 **±** 0.80^a^ (60.94%)	9.62 **±** 0.53^a^ (63.51%)
Group‐3 (100 mg/kg bw)	3.56 **±** 0.41^a,b^	5.39 **±** 0.58^c^ (22.63%)	4.67 **±** 0.56^c^ (21.20%)	4.39 **±** 0.26^b,c^ (10.93%)	4.14 **±** 0.17^b^ (15.22%)
Group‐4 (200 mg/kg bw)	3.62 **±** 0.58^a,b^	5.92 **±** 0.22^c^ (29.56%)	4.99 **±** 0.47^c^ (26.25%)	4.39 **±** 0.51^b,c^ (10.93%)	3.49 **±** 0.33^b^ (10.89%)
Group‐5 (400 mg/kg bw)	3.89 **±** 0.42^a^	9.19 **±** 0.81^b^ (54.62%)	6.99 **±** 0.85^b^ (47.35%)	5.31 **±** 1.11^b^ (26.37%)	4.16 **±** 0.49^b^ (25.24%)

*Note:* Experimental mean values are represented as mean ± SD (*n* = 6). Different letters of the data indicate different significance (*p* < 0.05; a < 0.05, b < 0.01, c < 0.001). For data analysis, Tukey's multiple comparison test was used.

## 4 Discussion

Phytochemical screening is useful to reveal the presence of phytoconstituent in plants that can be applied to predict the therapeutic efficacy of a plant (Pant et al. 2017). Some phytoconstituents are present in plants as secondary metabolites such as alkaloids, flavonoids, terpenoids, etc. that play a role in curing different diseases (Batiha et al. 2020). In a previous study, it was found that the root of *C. digyna* also contains alkaloids and flavonoids but terpenoid is absent (Tiranakwit et al. 2023). According to phytochemical screening in this research, different types of secondary metabolites were found that significantly increased the bioactivity of *C. digyna* leaves extract. Similar findings were also reported by other researchers that alkaloids have medicinal properties such as antipyretic, analgesic, and anti‐inflammatory effects (Alam et al. 2020). Tannins have astringent properties (tightening of tissues) and are also used for therapeutic purposes such as antioxidant, hypoglycemic, anticancer, and so on (Rajasekaran, Rajasekar, and Sivanantham 2021). Triterpenes are another type of compound that is known for their anti‐inflammatory as well as anti‐tumor properties (Renda, Gökkaya, and Şöhretoğlu 2022). Glycosides, especially cardiac glycosides, are useful for the treatment of heart diseases (Ayogu and Odoh 2020).

Brine shrimp lethality bioassay (BSLB) is a useful method to evaluate a variety of chemical compounds that are responsible for biological activities and demonstrate the relationship between cytotoxic and other therapeutic activities (Umaru et al. 2018). The positive control (vincristine sulfate) was significantly more lethal than the negative control (sea water), with an LC_50_ value of 1.61 μg/mL, while the LC_50_ value for leaves extract was 2.25 μg/mL. Based on the results of the BSLB, it can be enumerated that *C. digyna* leaves extract has a potent cytotoxicity compared to the positive control. If the LC_50_ value is greater than 1000 μg/mL in the BSLB of plant extract, it is considered safe (Khan et al. 2021). Based on the findings, our extract needs more safety tests which is the first priority for the formulation of any drug.

Accumulation of excessive fluid inside the membrane of HRBC results in rupture of the cell membrane which is known as hemolytic effect. Hypotonic solution induces the hemolysis of the membrane, and it causes secondary damage to the cell. This occurs through a chemical process called free radical lipid peroxidation which leads to the leakage of intracellular substances (Yesmin et al. 2020). Leakage is mediated by inflammatory mediators such as prostaglandins and leukotrienes (Arora and Ansari 2019). Phytochemicals can act as anti‐inflammatory agents by inhibiting the leakage of intracellular fluid (stabilization of HRBC membrane) (Yesmin et al. 2020). The *C. digyna* leaves extract showed 85.13% inhibition of HRBC membrane leakage which indicates significant anti‐inflammatory activity at high concentrations of 2000 μg/mL. This membrane stabilization activity was found in *C. digyna* leaves extract since it contained several phytochemicals such as flavonoids, alkaloids, terpenoids, or steroids. It was reported by other researchers that these phytochemicals are responsible for the stabilization effect of HRBC (Sonter et al. 2021).

Due to serious oxidative stress, the activity of several enzymes is altered. It also influences the occurrence of various diseases such as inflammation, diabetes, cancer, heart disease, rheumatoid arthritis, etc. Therefore, having antioxidant properties in a plant increases the possibility of anti‐inflammatory, antiarthritic, anti‐analgesic effects, and so on (Alam et al. 2013). Free radical DPPH was used to determine the scavenging effect of leaves extract to evaluate the antioxidant activity. At concentration of 500 μg/mL, leaves extract and AA showed 94% and 91% scavenging effect, respectively. With the increase of concentration of both leaves extract and AA, the percentage of this effect also increases that indicates, higher concentration of the leaves extract and AA contain more metabolites which causes the formation of DPPH‐H bonds to increase. As a result, the DPPH changes to a yellow color, which shows low absorbance and a high proportion of inhibition (Rohmah et al. 2020). Phenolic compounds in extract, such as flavonoids, triterpenes, and tannins, act as electron donors and donate electrons to DPPH which leads to the discoloration of the purple color of DPPH (Hasan et al. 2009; Rahman and Moon 2007). This color change indicates the presence of antioxidant activity in plant extract in several reports (Baliyan et al. 2022).

In protein denaturation, heat, external stress, and organic solvents like acid and base damage the structure of protein which produces auto‐antigens. The protein denaturation of bovine serum albumin may be due to the alteration of different bonds, for instance, hydrogen, electrostatic, disulfide, and hydrophobic bonds (Alamgeer, Uttra, and Hasan 2017; Rahman, Chinna Eswaraiah, and Dutta 2015). In this study, leaves extract showed concentration‐dependent inhibition of protein denaturation throughout the concentration range from low to high. In 1000 μg/mL, it has about 83.61% inhibition of protein denaturation whereas standard has 95.08% inhibition. So, the leaves extract of *C. digyna* can be used as a potent antiarthritic agent. The extract has the ability to inhibit this type of damage because of the presence of glycosides (Arya et al. 2011). Glycoside modifies protein by adding a sugar molecule causing stabilization of the protein (Jayaprakash and Surolia 2017).

Carrageenan‐induced edema is a nonspecific inflammation that depends on time and is biphasic. In the first phase, 0–1 h of carrageenan injection, the chemicals responsible for inflammation are histamine, serotonin, and bradykinin while in the later phase, after 1 h, involved mediators are prostaglandins, lysosome, and protease (Paramita et al. 2019; Sadeghi et al. 2014). Injection of polysaccharide carrageenan causes swelling of the paw in an animal model which is known as edema (Tasleem et al. 2014). At high doses the leaves extract reduces the inflammation rapidly, other doses also exert the same effect at a slow rate. The leaves extract inhibits this edema in the paw of mice which may be owing to the secondary metabolites of the leaves (Padmanabhan and Jangle 2012). Secondary metabolites exert this effect by inhibiting proinflammatory enzymes such as cyclooxygenase (COX) and lipoxygenase (LOX) or promoting anti‐inflammatory cytokines like IL‐10 and TGF‐β (Al‐Khayri et al. 2022; Mohammed et al. 2014). Regarding the anti‐inflammatory activity, it can be said that the extract has potent anti‐inflammatory as comparable to that of indomethacin.

In the acetic acid‐induced method, acetic acid causes the release of endogenous mediators like kinin, and prostaglandin, which produce pain in mice's abdomen (Gong et al. 2019). In the Tail immersion test, the leaves extract prolonged the stress tolerance capacity indicating the possible involvement of a higher center (Deshmukh and Sherkar 2019). The antinociceptive effect of leaves extract at the 400 mg/kg bw dose level was found to be significant with 54.62% elongation of reaction time in the tail‐immersion model as well as 52.74% inhibition of writhing in the acetic acid‐induced model. Thus, it appears the extract exhibits analgesia through peripheral and central (Hamann et al. 2019).

## 5 Conclusion

*Caesalpinia digyna* leaves extract contains various phytochemicals such as alkaloids, tannins, steroids, triterpenes, resins, glycosides, phenols, and carbohydrates. BSLB reveals that *C. digyna* leaves extract is free from danger to use as medication. Various in vitro and in vivo studies were performed and compared with control which revealed that it has significant anti‐inflammatory (The IC₅₀ value was 2.51 μg/mL in the in vitro test, and the in vivo test showed significance with *p* < 0.05), antiarthritic (83.61% inhibition at a dose of 1000 μg/mL), and antioxidant activity (IC₅₀ value was 0.13 μg/mL) as well as moderate analgesic activity (*p* < 0.05). Overall, these research findings suggest that *C. digyna* leaves have promising therapeutic potential for the treatment of various diseases and disorders, and further research is needed to explore its complete pharmacological profile and identify its active constituents.

## Author Contributions

**Kanij Fatema:** conceptualization (equal), data curation (equal), formal analysis (equal), investigation (equal), methodology (equal), validation (equal), writing – original draft (equal). **Tanzina Sharmin Nipun:** conceptualization (equal), methodology (equal), project administration (equal), supervision (equal), writing – original draft (equal), writing – review and editing (equal). **Md. Abdur Rashid Mia:** data curation (equal), formal analysis (equal), investigation (equal), methodology (equal). **S. M. Moazzem Hossen:** conceptualization (equal), investigation (equal), methodology (equal), project administration (equal), resources (equal), supervision (equal), validation (equal), writing – original draft (equal), writing – review and editing (equal).

## Ethics Statement

No test was conducted on the human body. In vivo test was performed in Swiss albino mice according to the rules of the Animal Ethics Review Board of the Faculty of Biological Science (Ref. No. AERB‐FBSCU‐20230731‐(1)).

## Conflicts of Interest

The authors declare no conflicts of interest.

## Data Availability

The data that support the findings of this study are available from the corresponding author upon reasonable request.
